# Crystal structure of 3-amino-2-ethyl­quinazolin-4(3*H*)-one

**DOI:** 10.1107/S2056989015014450

**Published:** 2015-08-06

**Authors:** Gamal A. El-Hiti, Keith Smith, Amany S. Hegazy, Mohammed Baashen, Benson M. Kariuki

**Affiliations:** a Cornea Research Chair, Department of Optometry, College of Applied Medical Sciences, King Saud University, PO Box 10219, Riyadh 11433, Saudi Arabia; b School of Chemistry, Cardiff University, Main Building, Park Place, Cardiff CF10 3AT, Wales; c Department of Chemistry, Faculty of Science and Humanities, Shaqra University, Al-Duwadmi, Saudi Arabia

**Keywords:** crystal structure, 3-amino-2-ethyl­quinazolin-4(3*H*)-one, π–π inter­actions

## Abstract

The mol­ecule of the title compound, C_10_H_11_N_3_O, is planar, including the ethyl group, as indicated by the N—C—C—C torsion angle of 1.5 (2)°. In the crystal, inversion-related mol­ecules are stacked along the *a* axis. Mol­ecules are oriented head-to-tail and display π–π inter­actions with a centroid-to-centroid distance of 3.6664 (8) Å. N—H⋯O hydrogen bonds between mol­ecules generate a ‘step’ structure through formation of an *R*_2_^2^(10) ring.

## Related literature   

For related compounds, see: Ma *et al.* (2013 ▸); Adib *et al.* (2012 ▸); Xu *et al.* (2012 ▸); Sasmal *et al.* (2012 ▸); Kumar *et al.* (2011 ▸); Rohini *et al.* (2010 ▸); Davies *et al.* (2010 ▸). For quinazolin-4(3*H*)-one ring-system modification through li­thia­tion, see: Smith *et al.* (2004 ▸, 1996 ▸, 1995 ▸). For the crystal structures of related compounds, see: El-Hiti *et al.* (2014 ▸); Yang *et al.* (2009 ▸); Coogan *et al.* (1999 ▸).
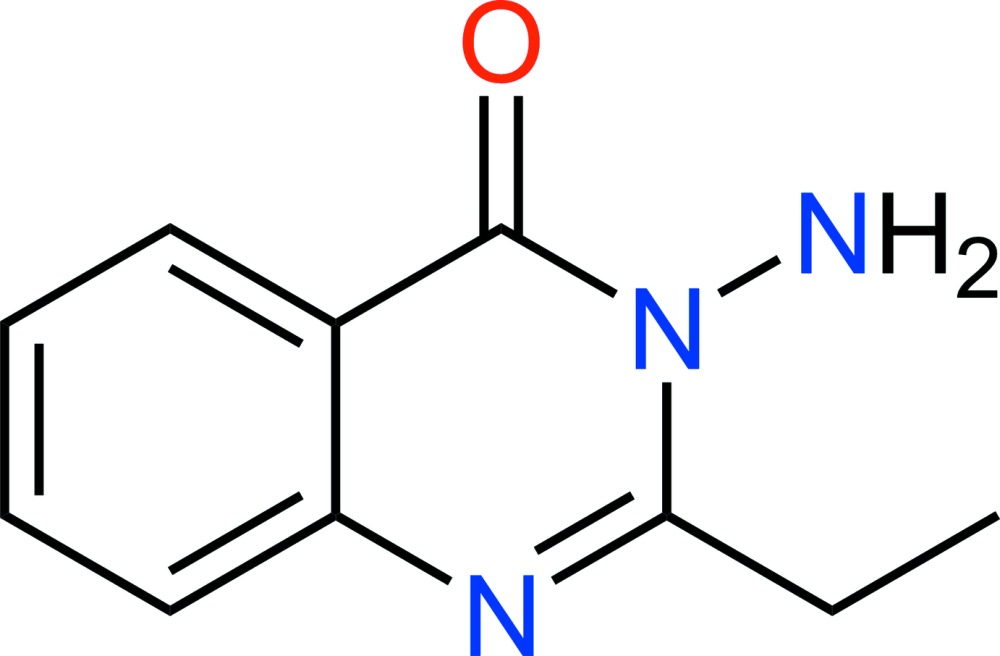


## Experimental   

### Crystal data  

C_10_H_11_N_3_O
*M* *_r_* = 189.22
Triclinic, 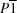

*a* = 7.0230 (5) Å
*b* = 7.6198 (7) Å
*c* = 9.7868 (6) Å
α = 69.709 (7)°
β = 89.242 (5)°
γ = 75.191 (7)°
*V* = 473.27 (7) Å^3^
*Z* = 2
Cu *K*α radiation
μ = 0.73 mm^−1^
*T* = 293 K
0.38 × 0.20 × 0.08 mm

### Data collection  

Agilent SuperNova Dual Source diffractometer with an Atlas detector
Absorption correction: Gaussian (*CrysAlis PRO*; Agilent, 2014 ▸) *T*_min_ = 0.741, *T*_max_ = 0.924
3303 measured reflections
1858 independent reflections
1657 reflections with *I* > 2σ(*I*)
*R*_int_ = 0.015
Standard reflections: 0

### Refinement  

*R*[*F* ^2^ > 2σ(*F* ^2^)] = 0.061
*wR*(*F* ^2^) = 0.196
*S* = 1.07
1858 reflections
136 parameters
H atoms treated by a mixture of independent and constrained refinement
Δρ_max_ = 0.35 e Å^−3^
Δρ_min_ = −0.22 e Å^−3^

### 

Data collection: *CrysAlis PRO* (Agilent, 2014 ▸); cell refinement: *CrysAlis PRO*; data reduction: *CrysAlis PRO*; program(s) used to solve structure: *SHELXS2013* (Sheldrick, 2008 ▸); program(s) used to refine structure: *SHELXL2013* (Sheldrick, 2015 ▸); molecular graphics: *ORTEP-3 for Windows* (Farrugia, 2012 ▸); software used to prepare material for publication: *WinGX* (Farrugia, 2012 ▸) and *CHEMDRAW Ultra* (Cambridge Soft, 2001 ▸).

## Supplementary Material

Crystal structure: contains datablock(s) I, New_Global_Publ_Block. DOI: 10.1107/S2056989015014450/hg5454sup1.cif

Structure factors: contains datablock(s) I. DOI: 10.1107/S2056989015014450/hg5454Isup2.hkl

Supporting information file. DOI: 10.1107/S2056989015014450/hg5454Isup3.cml

**Fig. 1.**
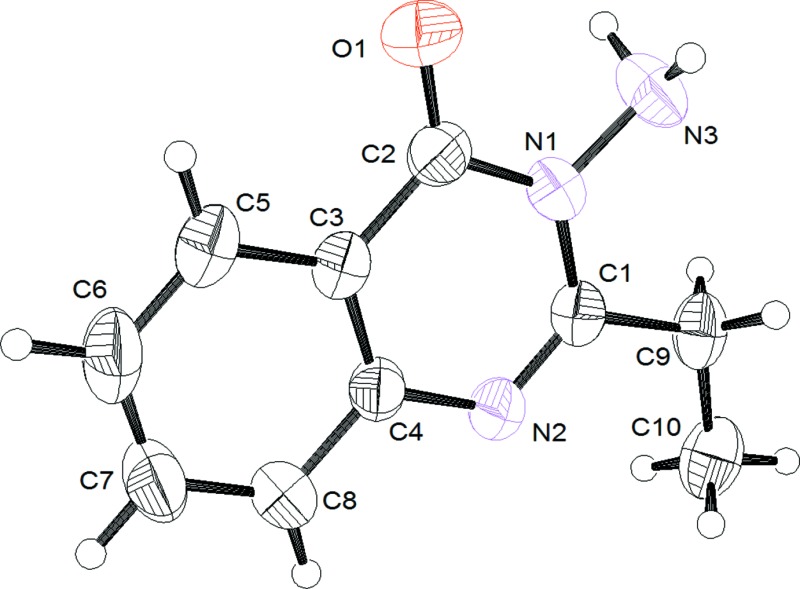
The asymmetric unit of C10H11N3O, with atom labels and 50% probability displacement ellipsoids for non-hydrogen atoms.

10 11 3 . DOI: 10.1107/S2056989015014450/hg5454fig1.tif
The asymmetric unit of C_10_H_11_N_3_O, with atom labels and 50% probability displacement ellipsoids for non-hydrogen atoms.

**Fig. 2.**
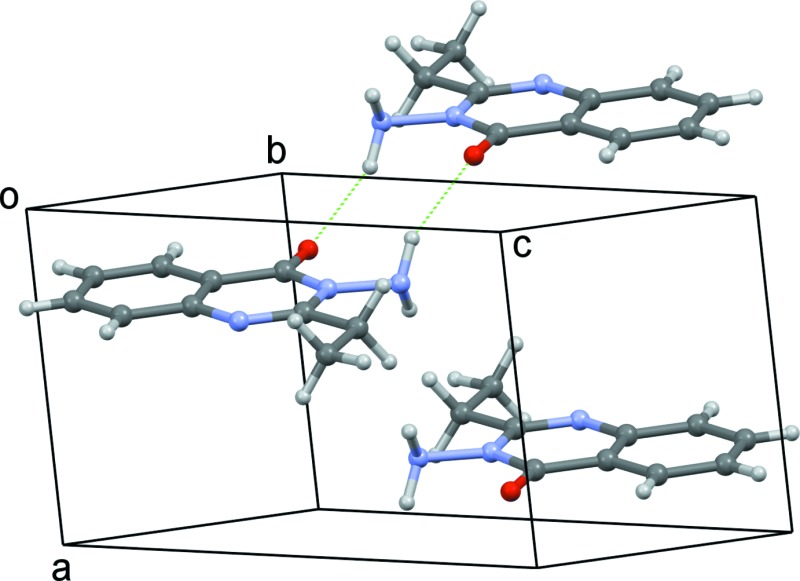
Crystal packing with hydrogen-bonding contacts shown as dotted lines.

. DOI: 10.1107/S2056989015014450/hg5454fig2.tif
Crystal packing with hydrogen-bonding contacts shown as dotted lines.

CCDC reference: 1416070

Additional supporting information:  crystallographic information; 3D view; checkCIF report

## Figures and Tables

**Table 1:** Hydrogen-bond geometry (, )

*D*H*A*	*D*H	H*A*	*D* *A*	*D*H*A*
N3H3*A*O1^i^	0.91(2)	2.12(2)	2.974(2)	157.1(19)

Symmetry code: (i) 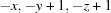.

## supplementary crystallographic information

### S1. Introduction

Quinazolines have various inter­esting biological applications (Sasmal *et al*., 2012; Rohini *et al.*, 2010). Quinazolin-4(3*H*)-ones synthesis involves use of various synthetic procedures. The most common starting materials are 2-amino­benzo­nitrile (Ma *et al.*, 2013), 2-bromo­benzamides (Xu *et al.*, 2012), isatoic anhydride (Adib *et al.*, 2012), anthranilic acid (Kumar *et al.*, 2011), methyl 2-amino­benzoate (Davies *et al.*, 2010). Li­thia­tion of 2-*n*-alkyl- and 2-unsubstituted 3-acyl­amino­quinazolin-4(3*H*)-ones with a lithium reagent in tetra­hydro­furan at a low temperature followed by reactions of various electrophiles with the lithium reagents produced in-situ gave the corresponding 2-substituted derivatives in good to excellent yields (Smith *et al.*, 2004, 1996, 1995). For the X-ray structures for related compounds, see: El-Hiti *et al.* (2014); Yang *et al.* (2009); Coogan *et al.* (1999).

### S2.1. Synthesis and crystallization

A mixture of methyl 2-amino­benzoate and propionic anhydride (1.4 mole equivalents) was heated for 30 minutes at 105 °C. The mixture was cooled to 75 °C and diluted with ethanol (50 mL). Hydrazine monohydrate (10 mole equivalents) was added in a dropwise manner over 10 minutes and the mixture was refluxed for 1 h. The mixture was cooled to room temperature and the solvent was removed under reduced pressure. The residue obtained was purified by column chromatography (silica gel hexane/di­ethyl ether in 4:1 by volume) to give 3-amino-2-ethyl­quinazolin-4(3*H*)-one in 82% yield (Davies *et al.*, 2010). Crystallization from a mixture of ethyl acetate and di­ethyl ether (1:2 by volume) gave colourless crystals of the title compound. The spectroscopic data for the title compound were identical with those reported (Davies *et al.*, 2010).

### S2.2. Refinement

H atoms were positioned geometrically and refined using a riding model with *U*_iso_(H) constrained to be 1.2 times *U*_eq_ for the atom it is bonded to except for methyl groups where it was 1.5 times with free rotation about the C—C bond. The amide hydrogen atoms were located in the difference Fourier map and refined freely.

### S3. Results and discussion

The asymmetric unit comprises a molecule of C_10_H_11_N_3_O (Fig. 1). The molecule is planar, including the ethyl group as indicated by the N2—C1—C9—C10 torsion angle of 1.5 (2)°. Inversion related molecules are stacked along the *a* axis (Fig. 2). Molecules (x,y,z) and (1-x, -y,1-z) are oriented head-to-tail and display π - π inter­action with a centroid to centroid distance of 3.66 (2)Å. N—H···O hydrogen bonds between molecules (x,y,z) and (-x,-y+1, -z+1) generate a 'step' structure through formation of a R_2_^2^(10) ring.

### Crystal data

**Table:**

C_10_H_11_N_3_O	*F*(000) = 200
*M**_r_* = 189.22	*D*_x_ = 1.328 Mg m^−^^3^
Triclinic, *P*1	Melting point: 398 K
*a* = 7.0230 (5) Å	Cu *K*α radiation, λ = 1.54184 Å
*b* = 7.6198 (7) Å	Cell parameters from 1907 reflections
*c* = 9.7868 (6) Å	θ = 6.5–73.7°
α = 69.709 (7)°	µ = 0.73 mm^−^^1^
β = 89.242 (5)°	*T* = 293 K
γ = 75.191 (7)°	Block, colourless
*V* = 473.27 (7) Å^3^	0.38 × 0.20 × 0.08 mm
*Z* = 2	

### Data collection

**Table:**

Agilent SuperNova Dual Source diffractometer with an Atlas detector	1657 reflections with *I* > 2σ(*I*)
ω scans	*R*_int_ = 0.015
Absorption correction: gaussian (*CrysAlis PRO*; Agilent, 2014)	θ_max_ = 73.9°, θ_min_ = 6.5°
*T*_min_ = 0.741, *T*_max_ = 0.924	*h* = −8→8
3303 measured reflections	*k* = −9→8
1858 independent reflections	*l* = −10→12

### Refinement

**Table:**

Refinement on *F*^2^	0 restraints
Least-squares matrix: full	Hydrogen site location: mixed
*R*[*F*^2^ > 2σ(*F*^2^)] = 0.061	H atoms treated by a mixture of independent and constrained refinement
*wR*(*F*^2^) = 0.196	*w* = 1/[σ^2^(*F*_o_^2^) + (0.1458*P*)^2^ + 0.021*P*] where *P* = (*F*_o_^2^ + 2*F*_c_^2^)/3
*S* = 1.07	(Δ/σ)_max_ < 0.001
1858 reflections	Δρ_max_ = 0.35 e Å^−^^3^
136 parameters	Δρ_min_ = −0.22 e Å^−^^3^

### Special details

**Table:**

Experimental. Absorption correction: CrysAlisPro, Agilent Technologies, Version 1.171.37.33 (release 27-03-2014 CrysAlis171 .NET) (compiled Mar 27 2014,17:12:48) Numerical absorption correction based on gaussian integration over a multifaceted crystal model Empirical absorption correction using spherical harmonics, implemented in SCALE3 ABSPACK scaling algorithm.
Geometry. All e.s.d.'s (except the e.s.d. in the dihedral angle between two l.s. planes) are estimated using the full covariance matrix. The cell e.s.d.'s are taken into account individually in the estimation of e.s.d.'s in distances, angles and torsion angles; correlations between e.s.d.'s in cell parameters are only used when they are defined by crystal symmetry. An approximate (isotropic) treatment of cell e.s.d.'s is used for estimating e.s.d.'s involving l.s. planes.

### Fractional atomic coordinates and isotropic or equivalent isotropic displacement parameters (Å^2^)

**Table:**

	*x*	*y*	*z*	*U*_iso_*/*U*_eq_	
C1	0.26318 (18)	−0.0469 (2)	0.59059 (14)	0.0441 (4)	
C2	0.18669 (19)	0.2641 (2)	0.38779 (16)	0.0485 (4)	
C3	0.21085 (18)	0.1504 (2)	0.29325 (14)	0.0455 (4)	
C4	0.25874 (19)	−0.0513 (2)	0.35723 (14)	0.0453 (4)	
C5	0.1873 (2)	0.2421 (3)	0.14073 (16)	0.0585 (4)	
H5	0.1547	0.3768	0.0991	0.070*	
C6	0.2123 (3)	0.1324 (3)	0.05335 (16)	0.0711 (5)	
H6	0.1976	0.1923	−0.0478	0.085*	
C7	0.2597 (3)	−0.0691 (3)	0.11692 (19)	0.0738 (5)	
H7	0.2761	−0.1428	0.0571	0.089*	
C8	0.2829 (3)	−0.1613 (2)	0.26567 (18)	0.0616 (5)	
H8	0.3144	−0.2961	0.3059	0.074*	
C9	0.2868 (2)	−0.1443 (2)	0.75358 (14)	0.0541 (4)	
H9A	0.1644	−0.0989	0.7931	0.065*	
H9B	0.3894	−0.1063	0.7928	0.065*	
C10	0.3393 (3)	−0.3628 (3)	0.80379 (17)	0.0703 (5)	
H10A	0.2385	−0.4018	0.7655	0.105*	
H10B	0.3492	−0.4162	0.9087	0.105*	
H10C	0.4636	−0.4096	0.7691	0.105*	
N1	0.21763 (16)	0.15389 (17)	0.53566 (13)	0.0475 (4)	
N2	0.28333 (17)	−0.14907 (17)	0.50721 (12)	0.0479 (4)	
N3	0.2043 (3)	0.2510 (2)	0.63756 (16)	0.0683 (5)	
O1	0.14455 (19)	0.44171 (16)	0.34527 (14)	0.0704 (4)	
H3A	0.080 (3)	0.329 (3)	0.630 (2)	0.079 (6)*	
H3B	0.292 (4)	0.328 (5)	0.609 (3)	0.111 (9)*	

### Atomic displacement parameters (Å^2^)

**Table:**

	*U*^11^	*U*^22^	*U*^33^	*U*^12^	*U*^13^	*U*^23^
C1	0.0404 (6)	0.0519 (7)	0.0392 (7)	−0.0129 (5)	0.0037 (5)	−0.0148 (5)
C2	0.0494 (7)	0.0445 (7)	0.0510 (8)	−0.0144 (5)	0.0072 (5)	−0.0151 (6)
C3	0.0457 (7)	0.0494 (8)	0.0400 (7)	−0.0154 (5)	0.0048 (5)	−0.0122 (6)
C4	0.0494 (7)	0.0498 (7)	0.0403 (7)	−0.0174 (5)	0.0067 (5)	−0.0173 (6)
C5	0.0588 (8)	0.0659 (9)	0.0424 (8)	−0.0201 (7)	0.0043 (6)	−0.0066 (6)
C6	0.0789 (11)	0.0986 (14)	0.0375 (7)	−0.0316 (10)	0.0073 (7)	−0.0206 (8)
C7	0.0926 (12)	0.0963 (13)	0.0520 (9)	−0.0368 (10)	0.0153 (8)	−0.0421 (9)
C8	0.0781 (10)	0.0627 (9)	0.0559 (9)	−0.0253 (8)	0.0121 (7)	−0.0312 (7)
C9	0.0482 (7)	0.0729 (9)	0.0367 (7)	−0.0158 (6)	0.0031 (5)	−0.0142 (6)
C10	0.0729 (10)	0.0711 (10)	0.0464 (8)	−0.0147 (8)	−0.0002 (7)	0.0010 (7)
N1	0.0511 (6)	0.0505 (7)	0.0451 (7)	−0.0128 (5)	0.0045 (4)	−0.0227 (5)
N2	0.0556 (7)	0.0458 (6)	0.0408 (7)	−0.0147 (5)	0.0055 (5)	−0.0129 (5)
N3	0.0801 (10)	0.0733 (9)	0.0628 (9)	−0.0136 (8)	0.0049 (7)	−0.0428 (7)
O1	0.0890 (8)	0.0436 (6)	0.0744 (8)	−0.0149 (5)	0.0118 (6)	−0.0182 (5)

### Geometric parameters (Å, º)

**Table:**

C1—N2	1.2937 (19)	C6—H6	0.9300
C1—N1	1.3840 (19)	C7—C8	1.370 (2)
C1—C9	1.4986 (18)	C7—H7	0.9300
C2—O1	1.2245 (18)	C8—H8	0.9300
C2—N1	1.3853 (19)	C9—C10	1.508 (2)
C2—C3	1.453 (2)	C9—H9A	0.9700
C3—C4	1.394 (2)	C9—H9B	0.9700
C3—C5	1.4027 (19)	C10—H10A	0.9600
C4—N2	1.3862 (18)	C10—H10B	0.9600
C4—C8	1.406 (2)	C10—H10C	0.9600
C5—C6	1.370 (3)	N1—N3	1.4227 (16)
C5—H5	0.9300	N3—H3A	0.91 (2)
C6—C7	1.392 (3)	N3—H3B	0.93 (3)
			
N2—C1—N1	122.58 (12)	C7—C8—H8	120.1
N2—C1—C9	120.35 (13)	C4—C8—H8	120.1
N1—C1—C9	117.07 (12)	C1—C9—C10	113.52 (13)
O1—C2—N1	120.90 (14)	C1—C9—H9A	108.9
O1—C2—C3	124.96 (14)	C10—C9—H9A	108.9
N1—C2—C3	114.14 (12)	C1—C9—H9B	108.9
C4—C3—C5	120.75 (14)	C10—C9—H9B	108.9
C4—C3—C2	118.64 (13)	H9A—C9—H9B	107.7
C5—C3—C2	120.61 (14)	C9—C10—H10A	109.5
N2—C4—C3	123.07 (12)	C9—C10—H10B	109.5
N2—C4—C8	118.33 (13)	H10A—C10—H10B	109.5
C3—C4—C8	118.60 (14)	C9—C10—H10C	109.5
C6—C5—C3	119.79 (16)	H10A—C10—H10C	109.5
C6—C5—H5	120.1	H10B—C10—H10C	109.5
C3—C5—H5	120.1	C1—N1—C2	123.65 (12)
C5—C6—C7	119.60 (14)	C1—N1—N3	117.72 (12)
C5—C6—H6	120.2	C2—N1—N3	118.63 (13)
C7—C6—H6	120.2	C1—N2—C4	117.90 (12)
C8—C7—C6	121.48 (16)	N1—N3—H3A	110.0 (14)
C8—C7—H7	119.3	N1—N3—H3B	104.8 (18)
C6—C7—H7	119.3	H3A—N3—H3B	109 (2)
C7—C8—C4	119.78 (16)		
			
O1—C2—C3—C4	−179.89 (12)	N2—C1—C9—C10	1.5 (2)
N1—C2—C3—C4	0.7 (2)	N1—C1—C9—C10	−179.13 (11)
O1—C2—C3—C5	0.4 (2)	N2—C1—N1—C2	0.9 (2)
N1—C2—C3—C5	−178.98 (10)	C9—C1—N1—C2	−178.40 (10)
C5—C3—C4—N2	−179.87 (11)	N2—C1—N1—N3	−178.35 (11)
C2—C3—C4—N2	0.4 (2)	C9—C1—N1—N3	2.30 (19)
C5—C3—C4—C8	0.0 (2)	O1—C2—N1—C1	179.18 (12)
C2—C3—C4—C8	−179.75 (11)	C3—C2—N1—C1	−1.4 (2)
C4—C3—C5—C6	−0.3 (2)	O1—C2—N1—N3	−1.5 (2)
C2—C3—C5—C6	179.44 (12)	C3—C2—N1—N3	177.87 (11)
C3—C5—C6—C7	0.4 (3)	N1—C1—N2—C4	0.3 (2)
C5—C6—C7—C8	−0.2 (3)	C9—C1—N2—C4	179.64 (10)
C6—C7—C8—C4	−0.1 (3)	C3—C4—N2—C1	−1.0 (2)
N2—C4—C8—C7	−179.95 (14)	C8—C4—N2—C1	179.19 (11)
C3—C4—C8—C7	0.2 (2)		

### Hydrogen-bond geometry (Å, º)

**Table:**

*D*—H···*A*	*D*—H	H···*A*	*D*···*A*	*D*—H···*A*
N3—H3*A*···O1^i^	0.91 (2)	2.12 (2)	2.974 (2)	157.1 (19)

Symmetry code: (i) −*x*, −*y*+1, −*z*+1.
