# Supplementary figures and images for: Crystal structure of 3-amino-2-ethyl­quinazolin-4(3H)-one
Source: Acta Crystallogr E Crystallogr Commun. 2015 Aug 6;71(Pt 9):o650–1. doi: 10.1107/S2056989015014450 (PMC4555403; doi:10.1107/S2056989015014450)

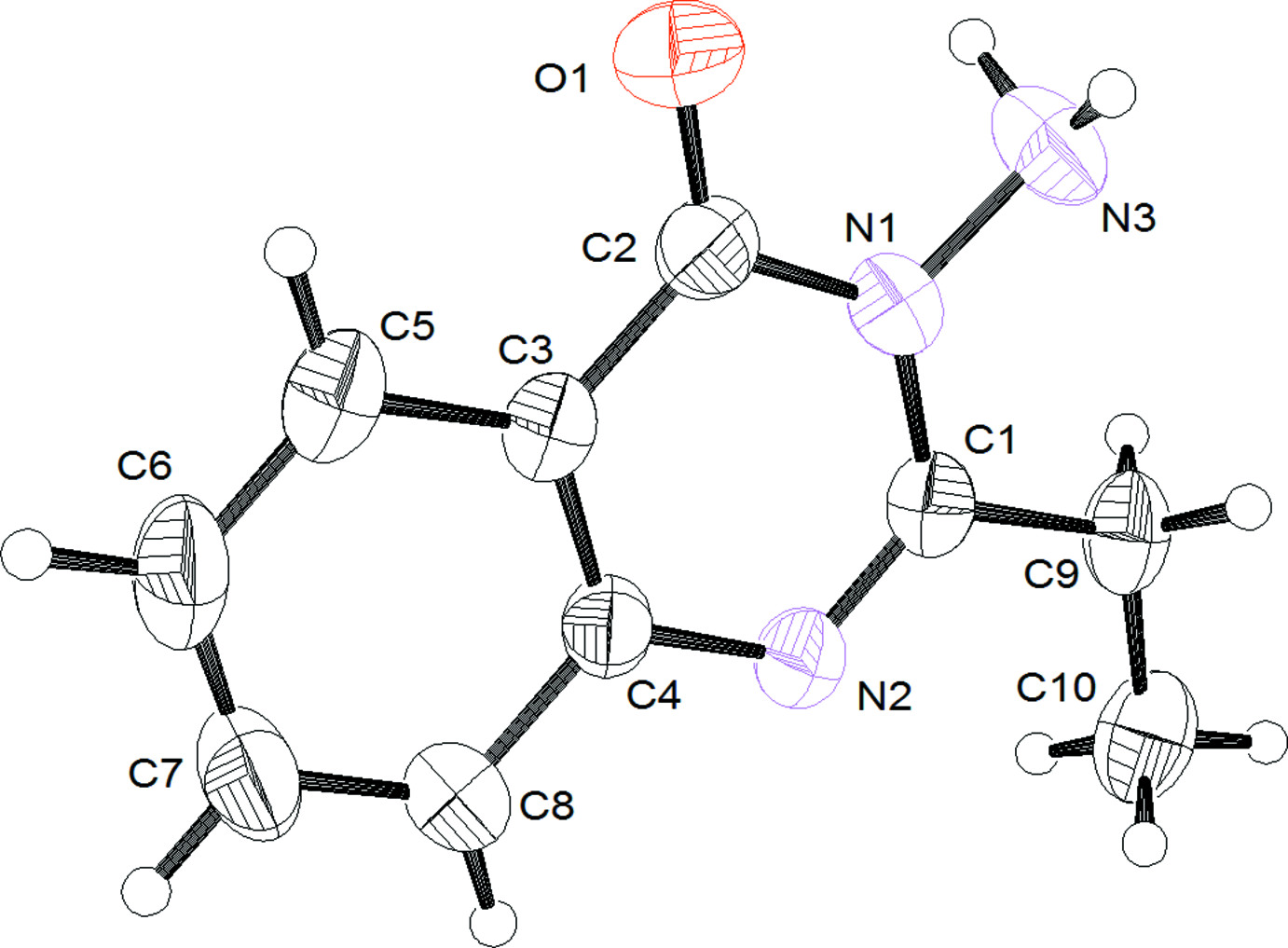

Supplement: Supplementary file 4 [file e-71-0o650-fig1.tif]

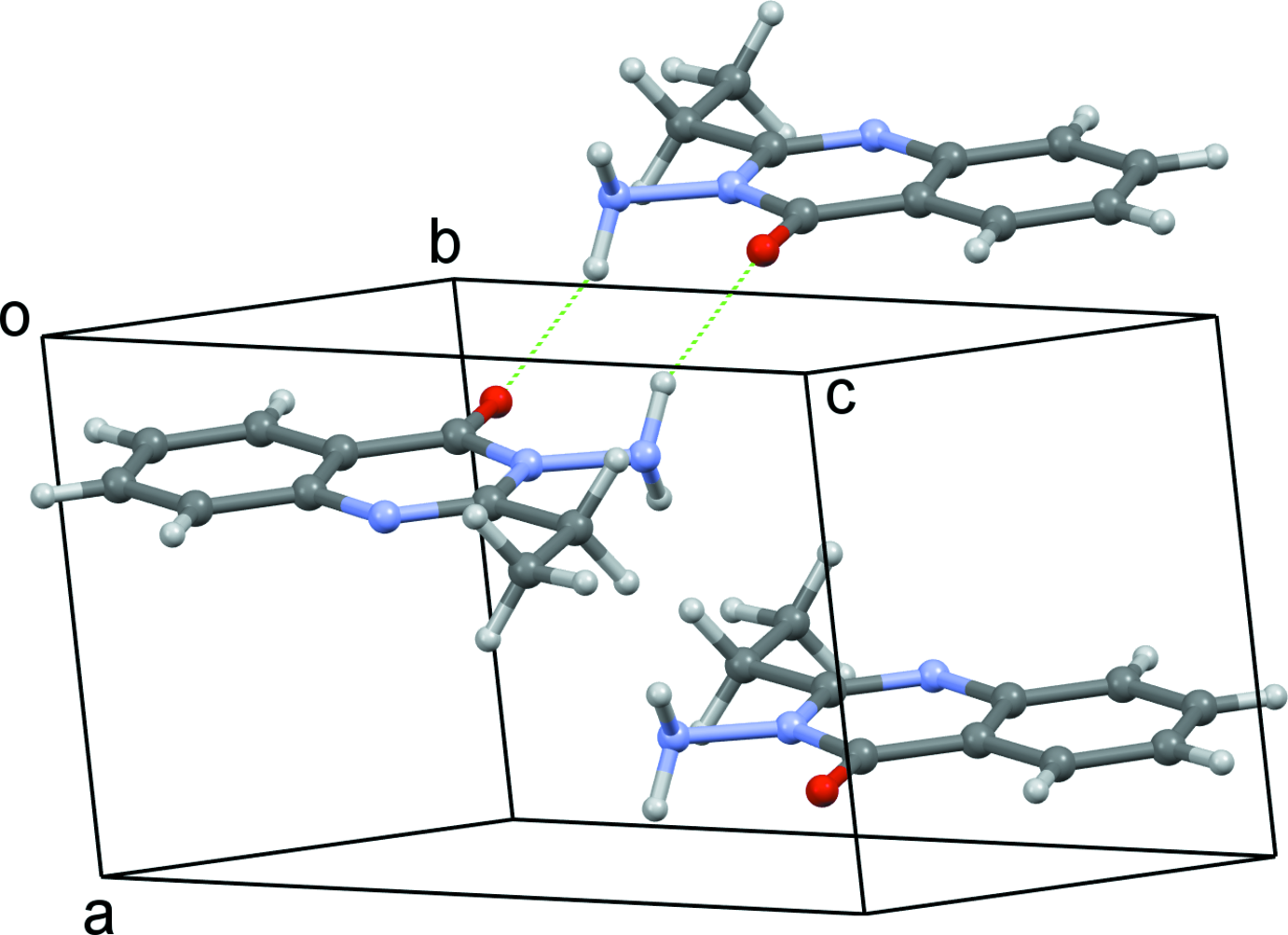

Supplement: Supplementary file 5 [file e-71-0o650-fig2.tif]
